# On the Use of Optically Stimulated Luminescent Dosimeter for Surface Dose Measurement during Radiotherapy

**DOI:** 10.1371/journal.pone.0128544

**Published:** 2015-06-08

**Authors:** Fasihah Hanum Yusof, Ngie Min Ung, Jeannie Hsiu Ding Wong, Wei Loong Jong, Vannyat Ath, Vincent Chee Ee Phua, Siew Ping Heng, Kwan Hoong Ng

**Affiliations:** 1 Department of Biomedical Imaging, Faculty of Medicine, University of Malaya, Kuala Lumpur, Malaysia; 2 Clinical Oncology Unit, Faculty of Medicine, University of Malaya, Kuala Lumpur, Malaysia; 3 University of Malaya Research Imaging Centre, Faculty of Medicine, University of Malaya, Kuala Lumpur, Malaysia; 4 Cancer Institute, Pantai Hospital Kuala Lumpur, Kuala Lumpur, Malaysia; China Medical University, TAIWAN

## Abstract

This study was carried out to investigate the suitability of using the optically stimulated luminescence dosimeter (OSLD) in measuring surface dose during radiotherapy. The water equivalent depth (WED) of the OSLD was first determined by comparing the surface dose measured using the OSLD with the percentage depth dose at the buildup region measured using a Markus ionization chamber. Surface doses were measured on a solid water phantom using the OSLD and compared against the Markus ionization chamber and Gafchromic EBT3 film measurements. The effect of incident beam angles on surface dose was also studied. The OSLD was subsequently used to measure surface dose during tangential breast radiotherapy treatments in a phantom study and in the clinical measurement of 10 patients. Surface dose to the treated breast or chest wall, and on the contralateral breast were measured. The WED of the OSLD was found to be at 0.4 mm. For surface dose measurement on a solid water phantom, the Markus ionization chamber measured 15.95% for 6 MV photon beam and 12.64% for 10 MV photon beam followed by EBT3 film (23.79% and 17.14%) and OSLD (37.77% and 25.38%). Surface dose increased with the increase of the incident beam angle. For phantom and patient breast surface dose measurement, the response of the OSLD was higher than EBT3 film. The *in-vivo* measurements were also compared with the treatment planning system predicted dose. The OSLD measured higher dose values compared to dose at the surface (Hp(0.0)) by a factor of 2.37 for 6 MV and 2.01 for 10 MV photon beams, respectively. The measurement of absorbed dose at the skin depth of 0.4 mm by the OSLD can still be a useful tool to assess radiation effects on the skin dermis layer. This knowledge can be used to prevent and manage potential acute skin reaction and late skin toxicity from radiotherapy treatments.

## Introduction

External beam radiotherapy (EBRT) is a treatment technique using high energy ionizing radiation which is used mainly for the treatment of cancers. In most clinics, routine quality assurances for machine output are generally in place to ensure the constancy of day to day radiation treatment delivery. However, as radiation therapy treatments become more complex, the role of supplementary dosimetry such as patient specific and *in-vivo* dosimetry are gradually gaining importance. The American Association of Physicists in Medicine (AAPM) Task Group (TG) 40 recommended that clinics should have access to thermoluminescence dosimeter (TLD) or other *in-vivo* dosimetry system in order to prevent major treatment errors [1]. During radiotherapy, the skin is at risk of skin toxicity such as erythema, necrosis, desquamation, dermal lymphatic and basal-cell carcinoma [2–4]. This is because while delivering a lethal dose to the tumour at a certain depth in tissue is of utmost importance, it is often done at the expense of the risk of developing skin reactions. The benefit of achieving a remission of the treated carcinoma certainly outweighs the risks. However, this does not make the management of patients developing tissue reactions any easier, particularly, if the patients have other medical conditions such as diabetes mellitus which may further complicate patient management [5]. Thus, understanding of the surface dose delivered to the surface of the skin may be useful in providing better clinical management of potential acute skin reactions. This can be done via in-vivo measurements, i.e with the dosimeter placed on the surface of skin, whreby the dose to a point inside the patient can be derived [6]. In addition, surface dose measurements can serve as a form of treatment verification to ensure the correct dose is being delivered during radiotherapy, in line with AAPM TG 40 recommendation.

Surface dose is defined as the dose deposited at the boundary between the air and the phantom [7]. It is contributed by scattered radiation from phantom, air and solid materials. Higher surface dose is deposited by increasing the oblique beam incidence and field size as well as usage of beam modifier devices such as the bolus, tray and immobilization device. [8–10]

Various groups have looked into the measurement of surface dose using different dosimeters. Quach et al. measured surface dose with radiochromic film, TLD and Metal Oxide Semiconductor Field Effect Transistor (MOSFET) using hemicylindrical solid water phantom simulating chest wall [11]. Meanwhile, Hsu et al. used Attix parallel-plate ionization chamber and TLD in measuring changes of surface dose as a function of bolus materials for conventional and intensity modulated radiation therapy (IMRT) [9]. By using various treatment parameters, Kry *et al*. compared the surface dose from TLD with Monte Carlo simulation [8]. The surface dose measurement had also been studied by Nakano et al. comparing Gafchromic EBT2 film and Attix parallel-plate ionization chamber measured surface dose on phantoms [12].

Various research groups have used different types of dosimeter to study surface dose, each having their own advantages and disadvantages. Radiochromic film is tissue equivalent and able to provide two-dimensional dose distribution but requires a waiting period of 24 hours after irradiation before the films can be scanned to allow for post-irradiation coloration to achieve stability [13, 14]. TLD is also near tissue equivalent but the reading process is very tedious and time consuming [15, 16]. Commercial MOSFET dosimeters provide real time feedback, but usually have a water equivalent depth (WED) of 0.8 mm to 1.8 mm, [17] which exceeds the recommended dosimeter thickness of 0.07 mm by the International Commission on Radiological Protection (ICRP) 1991 [18]. The use of the parallel-plate ionization chamber for in-vivo dosimetry is logistically impossible due to the curvature of the human body. In addition, it may require the application of many correction factors in high energy dosimetry [19–21]. To date, there is no ideal dosimeter that is suitable for in-vivo dosimetry. The choice of dosimeter is very much dependent on the availability of the dosimeter, its ease of use and the reliability of the dose measurements. The choice of dosimeter is also subject to the measurement purposes, whether two-dimensional results or point dose is needed, or if real time feedback is required to cater for treatment modifications.

Recently, optically stimulated luminescent dosimeter (OSLD), functioning as a passive dosimeter was commercialized as an in-vivo dosimeter. The physics of OSL dosimetry are somewhat similar to TL dosimetry except that it requires light photons to stimulate the release of dosimetric traps instead of heat. Readers are referred to Jursinic (2007) [22] for details of the OSLD physics.

In this work, we investigated the use of OSLD for surface dose measurement. The WED of the OSLD was first determined followed by surface dose measurement on a flat solid water phantom and an anthropomorphic phantom. The feasibility and accuracy of OSLD for clinical surface dose measurement was then conducted on a patient cohort undergoing breast radiotherapy.

## Materials and Methods

### The OSLD system

**Fig 1:**
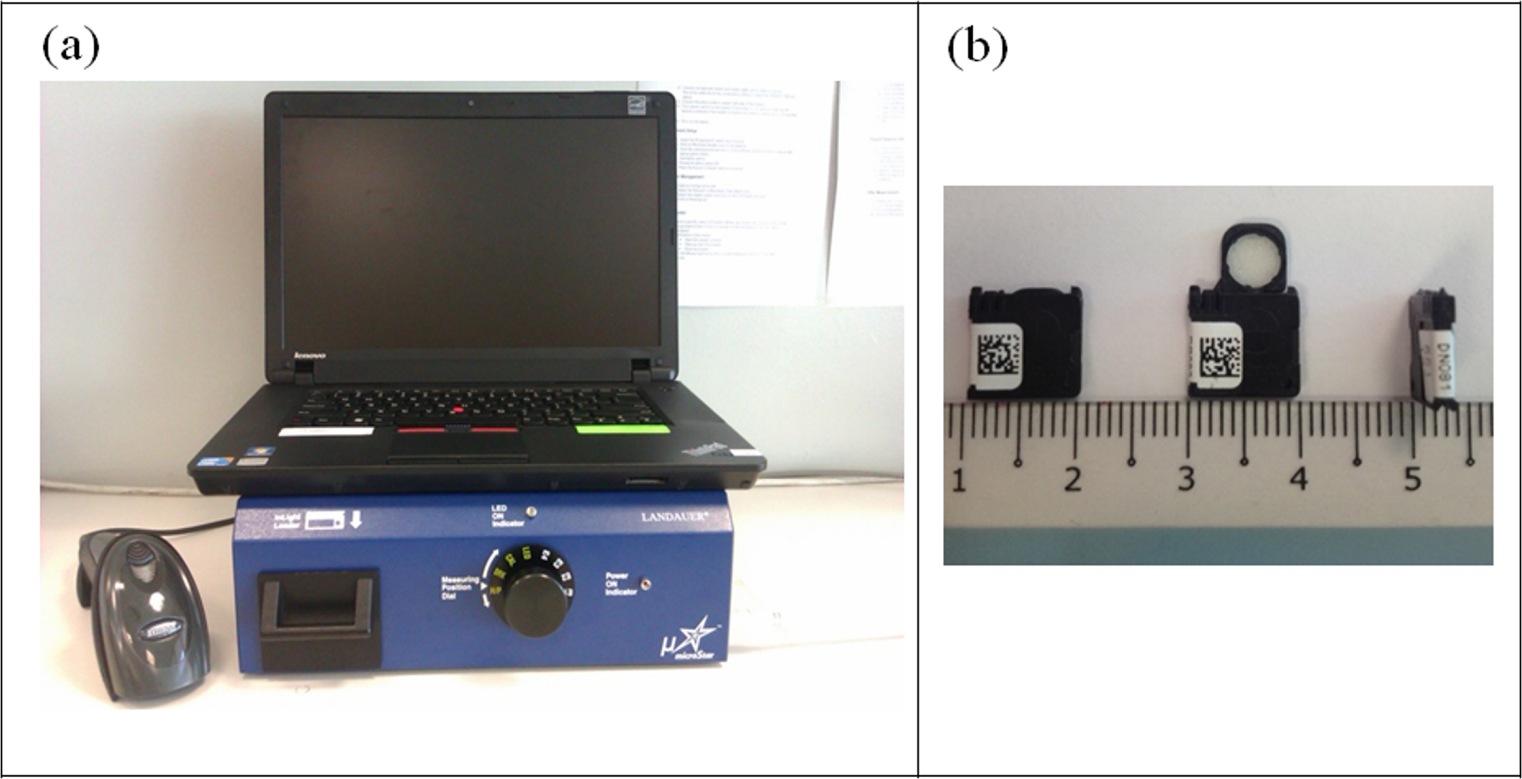
The MicroStar reader system and the nanoDot OSLD, respectively. The MicroStar reader system consists of a loader, a barcode scanner and a laptop.

The OSLDs used were nanoDot dosimeter (Landauer, Inc., Glenwood, IL) which are comprised of a 5 mm diameter disc Al_2_O_3_:C sensitive area encased in a light tight 1 cm × 1 cm × 0.2 cm plastic carrier. The dosimeter has a density of 1.03 g/cm^3^ (14). The OSLD were read using a microStar Reader (Landauer, Inc., Glenwood, IL). The microStar reader system and the nanoDot OSLD used in this study are shown in Fig 1(A) and 1(B), respectively. MicroStar software, version 4.3 was used to display and export the data in Microsoft Excel format for analysis. The post-irradiation reading was performed at least 30 minutes after irradiation for a stable number of counts [14, 22].

The OSLDs were first calibrated under a 6 MV photon beam using 30 cm × 30 cm × 20 cm solid water phantom with standard set up of 1.5 cm depth which is the depth of the maximum dose,(d_max_), 100 cm source-to-surface distance (SSD) and 10 cm × 10 cm field size. All measurements were carried out using a Varian Clinac 2100 C/D accelerator (Varian Medical System, Palo Alto, USA).

### The preparation of Gafchromic EBT3 film

Gafchromic EBT3 films (International Specialty Products, Wayne, NJ) were cut into squares of 2 cm × 2 cm^.^ The films were scanned 24 hours after irradiation using an Epson 10000XL flatbed scanner (Epson America, Inc. Long Beach, CA) to allow for post-irradiation color changes. The films were scanned using transmission mode, at a resolution and format of 96 dots per inch (dpi) and 48-bits RGB format, respectively. The scanned images were later saved as TIFF format to avoid compression and loss of data. The images were analyzed using ImageJ 1.47 software (National Institute of Health, USA). A set of standard EBT3 films were irradiated to establish the calibration curve.

### Water equivalent depth measurement

By using 20 cm of solid water phantoms as backscatter, 10 OSLDs were irradiated with 200 cGy dose using a 6 MV photon beam. The field size was set to 10 cm × 10 cm at 100 cm SSD. The OSLDs were irradiated one at a time by placing the dosimeters at the central axis of the beam on the surface of the solid water phantom.

Using the same set-up, a parallel-plate Markus ionization chamber (Markus type 23343 parallel plate ionization chamber, PTW Freiberg, Germany) was also used to measure surface and buildup doses. Sheets of 30cm × 30 cm Gafchromic XR-RV2 film (International Specialty Products, Wayne, NJ) with water equivalent thickness (WET) of 0.032 cm was used as buildup material. The WED of the film was calculated as the physical thickness of the film piece scaled by the physical density of the water (1.0g/cm^3^). Markus ionization chamber measurements were corrected for temperature and pressure, polarity and recombination. Correction factors for over-response were applied to the Markus ionization chamber readings using the formula by Gerbi and Khan (1990):

$$P’\left(d,E\right)=\;P\;\left(d,E\right)-\xi \left(0,E\right)le^{\alpha \left(d/d_{max}\right)}\;\left(\%\right)$$ (1)

ξ(0,E)=(−1.666+1.982IR)×(C−15.8)(%/mm) (2)

where *P* and *P’* are the corrected and uncorrected percentage depth doses in build-up region respectively, *ξ* (0,E) is the over-response of the chamber in percentage, at the surface of the phantom, *l* is the plate separation (2 mm for Markus PTW 23343), *α* is a constant with value 5.5, *d* is depth in phantom and *d*_*max*_ are 1.5 cm and 2.5 cm for 6 MV and 10 MV, respectively. IR represents the ionization ratio which is 0.664 and 0.732 for 6 MV and 10 MV, respectively using the specific setup in this study and *C* is the sidewall-collector distance (0.35 mm for Markus PTW 23343). The calculated *ξ (0*,*E)* are respectively 5.407 and 3.324 for 6 MV and 10 MV photon beams, respectively.

The WED of OSLD was investigated by comparing the surface dose of the OSLD with the measurement using the Markus ionization chamber converted to percentage depth dose (PDD).

### Surface dose measurement on solid water phantom

Surface dose measurement was performed by using three different dosimeters namely the Markus ionization chamber, EBT3 film and OSLD on a solid water phantom. The dosimeters were placed on a 30 cm × 30 cm × 20 cm solid water phantom, at the central axis of 10 cm × 10 cm radiation field with SSD of 100 cm. A dose of 200 cGy was delivered to each dosimeter, one at a time for photon energies of 6 MV and 10 MV. The measurements were repeated twice for each dosimeter. Absorbed doses recorded by all dosimeters were normalized to the 100% dose at d_max_.

### Effect of incident beam angle on surface dose

An OSLD was placed at the central axis on the surface of a 30cm × 30cm × 20 cm solid water phantom and irradiated using 6 MV photon beam under standard set-up (100 cm SSD and 10 cm × 10 cm field size). The OSLD response for different incident beam angles was evaluated by delivering a fixed number of MUs, with the linear accelerator gantry rotated to an angle. The incident beam angles of -75° to +75°, with increment of 15° were studied. The responses of the OSLDs were compared with EBT3 film measurements using the same experimental setup. The measurements were repeated twice for each dosimeter

### Surface dose measurement on anthropomorphic phantom

Prior to actual application of the OSLD on clinical patients, simulated breast radiotherapy treatments were carried out on a female anthropomorphic phantom (Atom Phantom, CIRS, Norfolk, VA). Eclipse Treatment Planning System (TPS) (Varian Medical System, Palo Alto, USA) version 10.0 was used to create (i) a chest wall irradiation (with bolus) and (ii) a breast conserving (without bolus) radiotherapy treatment plans on CT images of the anthropomorphic phantom. The TPS used a pencil beam convolution (PBC) algorithm with a grid size of 2.5 mm x 2.5 mm. Two tangential opposed beams were used with different energy weighting and beam modifier devices such as the physical wedge. The chest wall surface dose measurements were carried out on the anthropomorphic phantom without breast attachment while breast conserving radiotherapy was carried out with breast attachment. During the measurement, the OSLDs were placed at 3 cm from the border of the medial and lateral beams [23]. The contralateral dose was measured by placing the dosimeter on the tip of the nipple of the contralateral breast. The positions of the dosimeters were fixed by putting a marker on the surface for good reproducibility and accuracy. For chest wall measurements, the dosimeters were placed underneath the 1 cm thickness bolus. The response of the OSLD was compared with the measurements by EBT3 film and the dose predicted by Eclipse TPS. The measurements were repeated twice for each dosimeter

### Surface dose measurement: Patient study

For surface dose measurement on patients, 5 patients undergoing breast conserving radiotherapy and 5 patients undergoing chest wall irradiation were recruited. The placement of the OSLD was the same as described in the anthropomorphic phantom study. Measurements were repeated at least twice during different treatment fractions throughout the whole course of breast radiotherapy. The OSLD measurements were also compared with EBT3 film measurements and TPS predicted dose. A total dose of 40 Gy dose was prescribed for 15 fractions with a single prescribed dose of 2.67 Gy being delivered for each treatment fraction. The Planning Target Volume coverage was planned to receive 95% to 107% of the prescribed dose [24]. The OSLD measurements were compared with TPS predicted doses and EBT3 film measurements using paired sample t-test by IBM SPSS Statistics software for Windows (IBM SPSS, IBM, New York, USA) Version 21. The medical ethics was approval was given by the Medical Ethics Committee of University Malaya Medical Centre (UMMC) (Reference number: 1030.19). Written consent was waived, as the study did not involve the use of drugs and patient intervention. Verbal consent to the patient, a method approved by the medical ethics committee, was conducted before the radiotherapy treatment.

## Results

### WED of OSLD

The WED of the OSLD was determined by substituting the percentage of normalized surface dose to the dose at d_max_ measured by the OSLD into the equation by the PDD graph of Markus ionization chamber’s measurements. The WED of the OSLD was found to be 0.4 mm.

### Surface dose measurement using flat solid water phantom

Table 1 shows the normalized response of the Markus ionization chamber, EBT3 film and OSLD for 6 MV and 10 MV photon beams. The surface dose recorded for 6 MV photon measured by the detectors were higher than that of the 10 MV photon due to the skin sparing effect of 10 MV photon. The Markus ionization chamber measured the lowest surface dose, followed by EBT3 film and OSLD. The differences between the three detectors were larger for the lower beam energy. This response was expected as the dose gradient in the buildup region for 6 MV photon beam is steeper than that of the in 10 MV photon beam.

**Table 1:** Surface dose (normalized to 100% the dose at d_max_) of different dosimeters for 6 MV and 10 MV photon beams.

Energy	Mean surface dose ± 1 s.d (%)		
	Markus	EBT 3 Film	OSLD
**6 MV**	15.95 ± 0.08	23.79 ± 0.68	37.77 ± 2.00
**10 MV**	12.64 ± 0.03	17.14 ± 1.68	25.38 ± 0.30

The measured WED and surface doses of Markus ionization chamber, EBT3 film and OSLD were compared to the measurements by Jong *et al*. which used MO*Skin* detector and EBT2 film. The MO*Skin* detector and EBT2 film has WED of 0.070 mm [25] and 0.122 mm, respectively [26].

**Fig 2:**
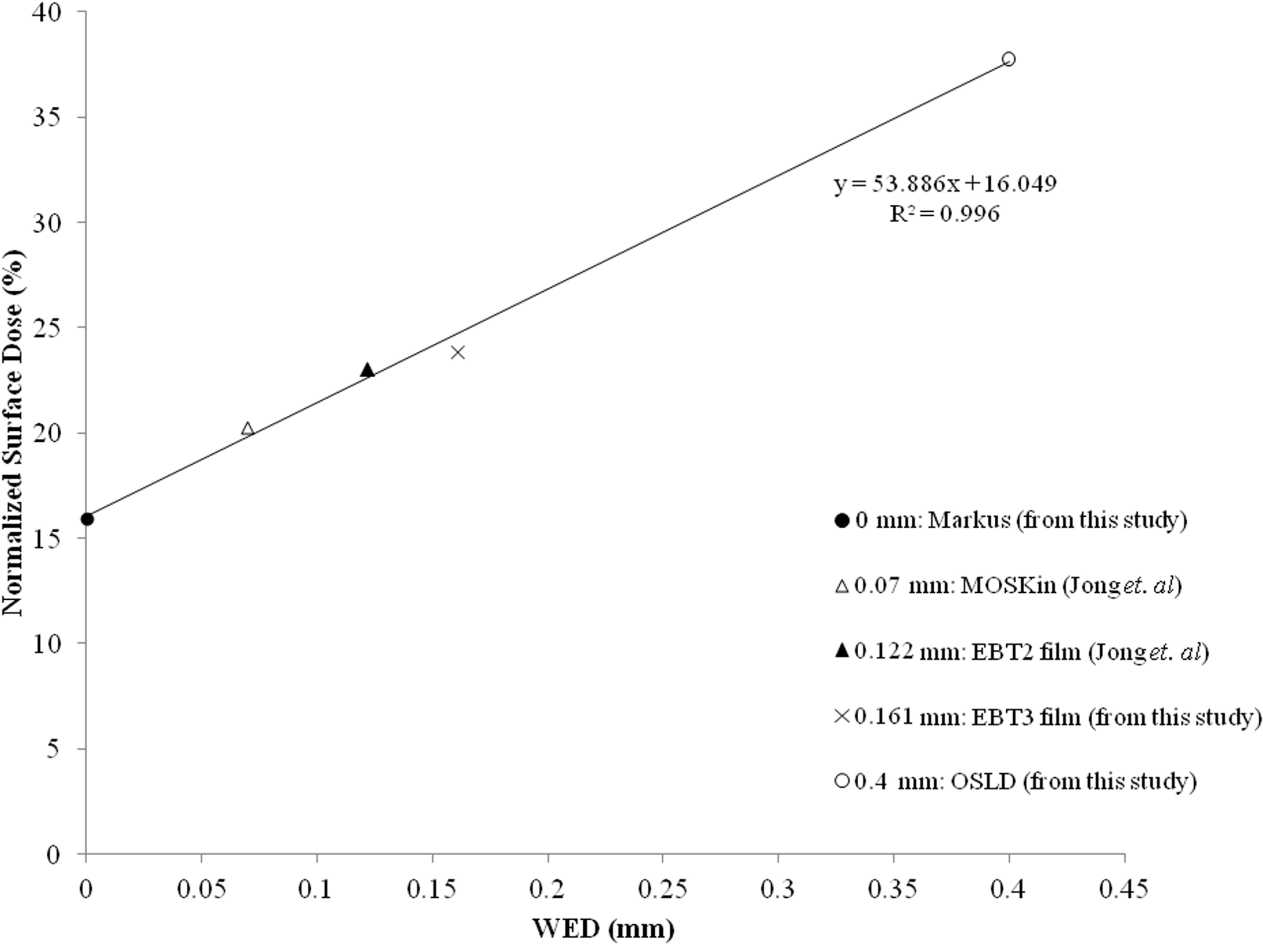
Normalized surface dose of dosimeters with different WED. The graph shows the relationship of the WED with the surface dose measured.

The relationship between the WED and surface dose is shown in Fig 2. The increase in WED which is the intrinsic buildup of the dosimeter, led to the increase in measured surface dose. The data points were fitted in a simple linear trend line (R^2^ = 0.996). An interpolation method was used to determine the correction factors of the OSLD for surface dose (Hp(0.0)) and skin dose (Hp(0.07)). *In-vivo* OSLD measurements can be used to predict surface dose (Hp(0.0)) and skin dose (Hp(0.07)) by means of applying a correction factor using the following equations.

$$Surface\;dose,\;Hp\left(0.0\right)=\;D_{OSLD}\;\times 0.42$$

$$Skin\;dose,\;Hp\left(0.07\right)=\;D_{OSLD}\;\times 0.54$$

### Effect of incident beam angle on surface dose

**Fig 3:**
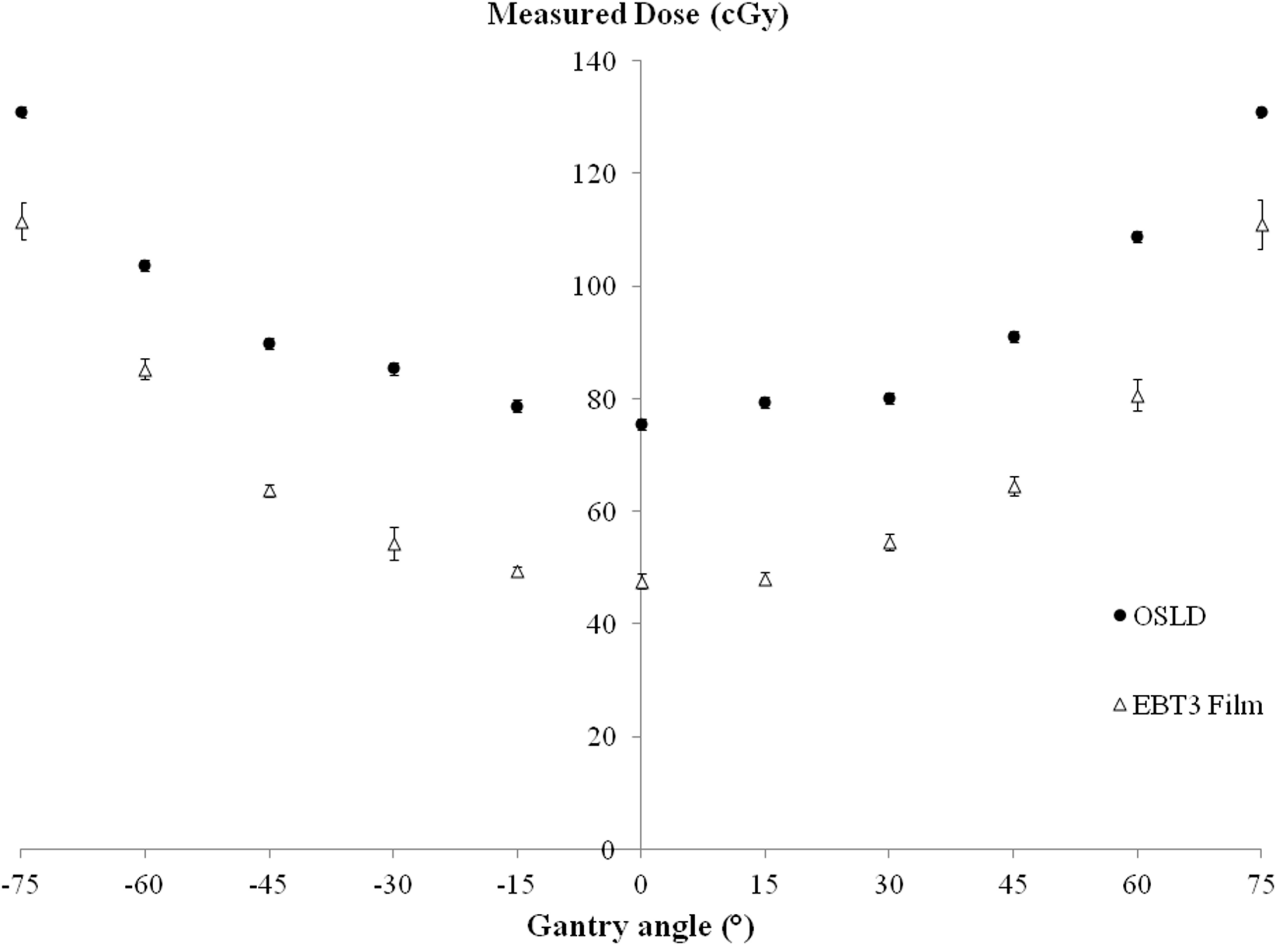
Measured surface dose for various incident beam angles measured on a flat surface. Relationship between the beam angle and surface dose measured. The error bars represent one S.D. of the average of at least 3 repeated surface dose measurements.

Fig 3 shows the effect of incident beam angle on surface dose. The error bars represent 1 SD of the mean of three OSLD measurements. As the beam incident angle increases, the measured surface dose increased as a function of inversed cosine. For small incident beam angles (θ ≤ 30°), the measured surface dose increased by less than 10%. At the maximum incident beam angles of 75°, a 73% increase in the surface dose was found when measuring using OSLD. This may be due to the shift of the region of charged particle equilibrium toward the surface.

Compared to EBT3 film measurements, the dose recorded by the OSLD is higher with average percentage difference of 39.1 ± 17.5%. This is due to the different WED of the dosimeters. Both dosimeters showed a similar trend, showing an increase in the measured dose with increased incident beam angles. However, the EBT3 film measurements showed a steeper curve compared to the OSLD. This may be because of the slight angular dependence of the nanoDot packaging design. Kern et al. measured the angular dependence of the nanoDot OSLD in a cylindrical phantom [27]. They reported the angular dependence of the nanoDot detector to be <4%.

### Surface dose measurement on anthropomorphic phantom

Table 2 shows the surface dose measured on an anthropomorphic phantom. The OSLD and EBT3 film measurements were compared with the TPS predicted dose. In general, the OSLD recorded higher dose compared to EBT3 film for all exposures. The TPS appears to predict slightly higher dose for the treated breast and much lower dose were predicted for the contralateral breast.

**Table 2:** Surface dose measured using OSLD and EBT3 film during breast conserving and chest wall radiotherapy compared with the TPS predicted dose.

Type of Irradiation	Energy	Dosimetry	Mean Surface Dose ± 1 s.d (cGy)		
			Medial	Lateral	Contralateral
**Breast Conserving**	**6 MV**	**TPS**	137.0 ± 1.7	159.7 ± 2.6	4.4 ± 0.0
		**EBT3**	116.2 ± 1.0	123.7 ± 1.6	18.8 ± 0.6
		**OSLD**	138.0 ± 6.9	144.7 ± 3.3	22.2 ± 1.7
	**10 MV**	**TPS**	125.3 ± 1.9	131.8 ± 0.1	3.6 ± 0.0
		**EBT3**	113.0 ± 1.5	118.1 ± 1.3	20.2 ± 2.7
		**OSLD**	118.6 ± 0.6	131.9 ± 1.9	21.7 ± 0.9
**Chest Wall**	**6 MV**	**TPS**	273.4 ± 1.3	274.5 ± 0.0	2.8 ± 0.0
		**EBT3**	248.8 ± 2.4	247.4 ± 2.7	18.3 ± 0.1
		**OSLD**	265.1 ± 0.6	256.5 ± 2.4	19.0 ± 0.8
	**10 MV**	**TPS**	269.7 ± 0.2	259.8 ± 0.1	3.2 ± 0.0
		**EBT3**	251.4 ± 2.5	247.7 ± 1.5	21.0 ± 1.8
		**OSLD**	264.4 ± 3.0	254.0 ± 2.0	22.3 ± 0.5

For breast conserving radiotherapy, the average measured surface doses were found to range from 44.4% to 54.2% of the prescribed dose (267 cGy). Under 6 MV photon beam, OSLD measurements were higher than EBT3 by a factor of 1.19 and 1.17 for medial and lateral positions, respectively. The doses recorded from OSLD measurements were comparable to, or slightly lower than the TPS predicted doses. For the treatment plans delivered using 10 MV photon beams, the OSLD measurements were also higher than the EBT3 film measurements, although by a lesser degree. The OSLD measurements were also comparable to the TPS predicted dose for this beam energy.

For chest wall radiotherapy, the average measured surface doses were found to range from 95.1% to 99.3% of the prescribed dose. The differences between the OSLD and EBT3 film measurements were also found to be smaller compared to breast conserving radiotherapy. For medial and lateral positions, the OSLD measurements were found to be higher than EBT3 film by a factor of 1.07 and 1.04, respectively for 6 MV photon beams. Using the same energy, the OSLD recorded lower surface dose as compared to the TPS predicted dose by a factor of 0.97 for medial position and 0.93 for lateral position. By using 10 MV photon beams, the OSLD measured a higher surface dose than EBT3 film for the medial and lateral positions of the treated breast, and a slightly lower dose as compared to the TPS predicted dose.

For surface dose of the contralateral breast, the OSLD and EBT3 film measurements were in close agreement with each other. However, large discrepancies were observed between the measurements and the TPS predicted dose values. The TPS predicted dose was less than 5 cGy for all measurement points whereas direct measurements showed that the actual surface dose to the contralateral breast could be more than 18 cGy. This represents 6.7% of the predicted dose.

### Surface dose measurement: Patient study

**Fig 4:**
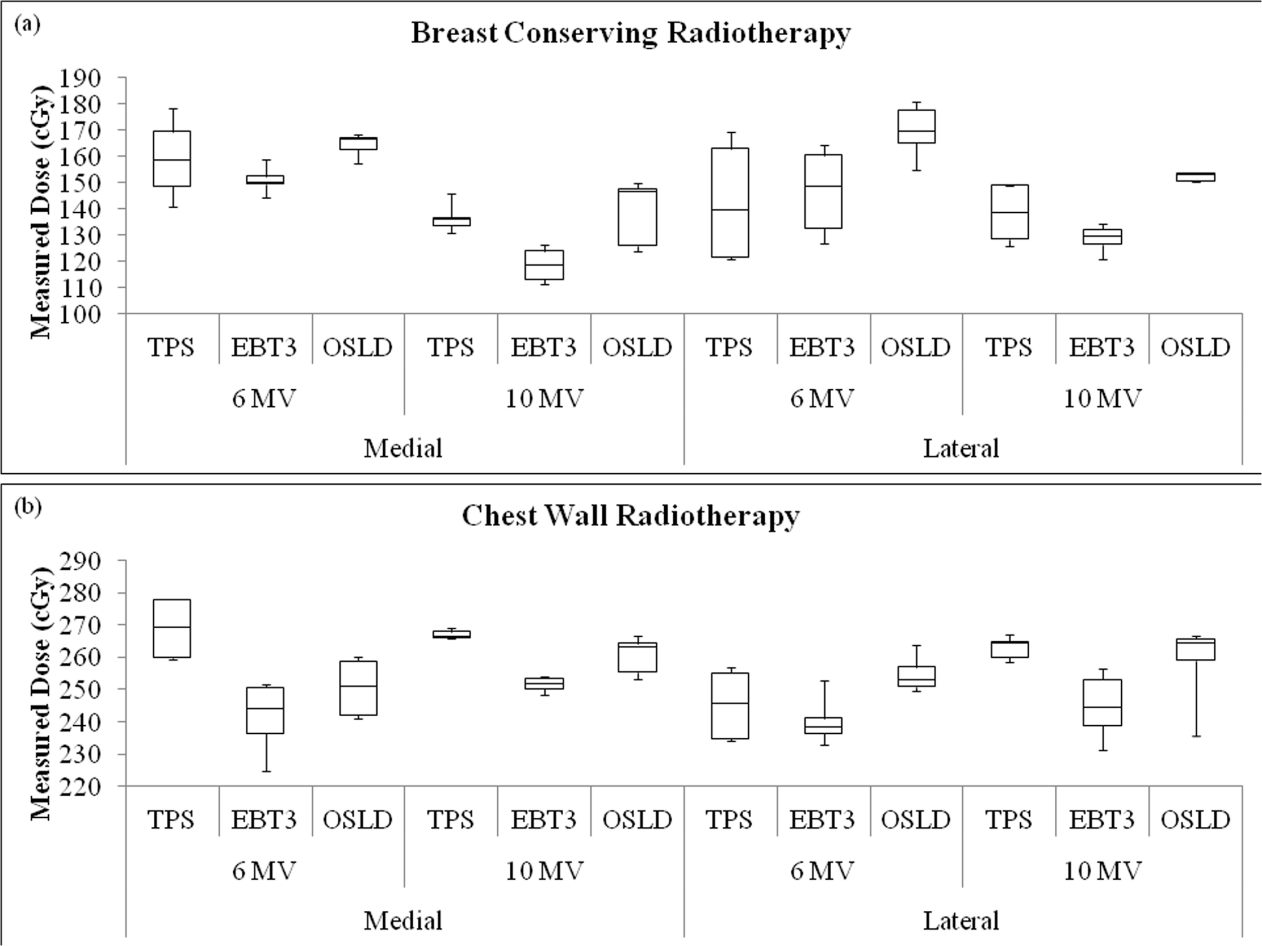
The graphs of surface dose measured on the treated breast. The surface dose of the treated breasts was measured for medial and lateral positions during breast conserving and chest wall radiotherapy, respectively.

The *in-vivo* dose measurements of 5 breast conserving and 5 chest wall radiotherapy patients are shown in Fig 4(A) and 4(B), respectively. Each box-plot represents the spread of 15 measurements (5 patients × 3 measurements). For breast conserving radiotherapy, the difference between the three measurements for the same patients was found to be ±7.71 cGy, ±4.14 cGy and ± 2.18 cGy for medial, lateral and contralateral positions, respectively. For chest wall radiotherapy, the differences were within ±7.11 cGy, 8.55 cGy and ±1.72 cGy for medial, lateral and contralateral positions, respectively.

The box-plots shows a similar trend for both breast conserving and chest wall radiotherapy, whereby the median doses measured by the OSLD were higher than those measured using EBT3 films. The small spread of the box-plots shows that the *in-vivo* measurements were quite consistent. However, day-to-day patient positioning and set-up uncertainty could contribute to the measured dose distributions. The box-plots for TPS predicted dose values appear to have a larger spread. This may be due to the variability in the point of measurement and the larger dose calculation grid size. Chest wall radiotherapy also showed higher surface dose compared to breast conserving radiotherapy. This was due to the application of 1.0 cm bolus which removes the skin sparing effect and shifted the target volume to the skin. There was a statistical significant difference (99% CI) in comparison of OSLD measurements (N = 90) with TPS predicted dose (N = 90; p<0.01) and EBT3 film measurements (N = 90; p<0.01).

**Fig 5:**
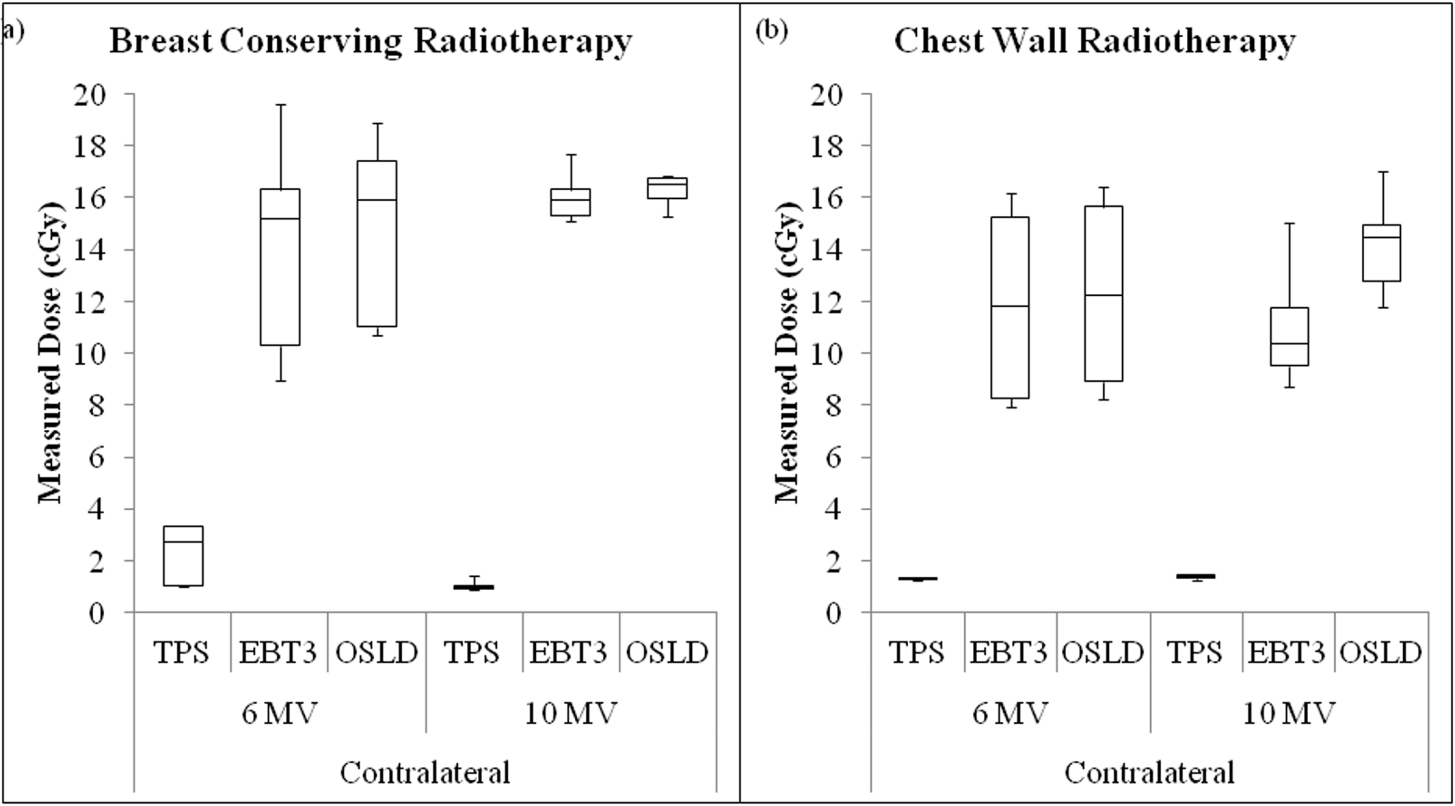
The surface dose measured by the contralateral breast. The surface dose of the contralateral breast was measured for breast conserving and chest wall radiotherapy, respectively.

Fig 5 shows the box-plot of the surface dose measured on the contralateral breast of the 10 patients. The box-plot shows a similar trend to the phantom study. *In-vivo* measurements using OSLD and EBT3 film measurements were found to be in good agreement with each other albeit much higher than those predicted by the TPS. Median surface dose measured was higher than 14 cGy and 10 cGy for breast conserving and chest wall radiotherapy, respectively. In comparison, the TPS predicted a median dose of <3 cGy. This may be because the TPS does not take into account absorbed dose due to scattered radiation. The scattered radiation from the treated breast to the contralateral breast could be as high as 18.87 cGy per fraction.

## Discussion

The work of identifying the suitability of the OSLD to be used for surface dose measurement started with determining the WED of the OSLD by comparing the surface dose measured by the OSLD to the depth dose measurement made using a Markus ionization chamber [28, 29]. The OSLD was found to have a WED of 0.4 mm which consequently over-estimated the surface dose by 21.8% for the 6 MV photon beam. This means that OSLD is neither measuring surface dose at a depth of 0 mm (Hp(0.0)), nor measuring skin dose (Hp (0.07)) at a depth of 0.07 mm [18]. ICRP defined skin dose at the basal skin layer, which is considered as the most radiosensitive layer at the depth of 0.07 mm in the skin [18]. However, anatomically, human skin consists of two layers which are the epidermis and dermis layers. The epidermis and dermis layers are physically 2.7 mm in thickness [30, 31]. Thus, the OSLD which has a WED of 0.4 mm can still be considered to measure the dose to the skin layer, albeit at a greater depth than the basal skin layer. Using OSLD measurements, it is possible to estimate the dose to the surface of the skin or the basal skin layer Hp (0.07) by means of a correction factor. However, one should note that this correction factor can be applied for perpendicular beam incidence only.

The measurement of surface dose on a flat solid water phantom showed that the dosimeter with less intrinsic build-up measured lower dose. EBT3 film has an active layer of 17 μm and density of 1.1 g/cm^3^ that is structurally sandwiched between two layers of transparent polyester with thickness of 97 μm [7]. It has an intrinsic buildup of 0.16 mm. On the other hand, the parallel-plate Markus ionization chamber which has an entrance electrode window thickness scaled by the physical density of the electrode material, corresponds to the effective point at a depth [20] which is the water equivalent depth at surface. The increase of buildup which can be expressed in water equivalent depth led to the increase of dose measured [26]. It has been shown by Devic *et al*. that the PDD increased exponentially from 14% at depth of 4 μm to 43% at a depth of 1 mm [7].

Oblique incident beam angles are expected to contribute to higher surface dose due to the shift of the charged particle equilibrium region to the surface as well as increase in electron contaminations and higher photon interactions within the phantom [32]. All of these factors resulted in the combined effect of increased surface dose measured, particularly during tangential breast radiotherapy treatments in which the range of the gantry angle used was 49° to 58° positively or negatively from the y-axis.

The feasibility of the OSLD for measuring surface dose was demonstrated in an anthropomorphic phantom study as well as clinical measurements. Small standard deviations reflected good reproducibility of the dosimeter’s response and exposure setup. Based on the presented results, it had been demonstrated that OSLD recorded higher surface dose as compared to EBT3 film responses for all positions in phantom and patient studies. This was due to the difference in the WED of the dosimeters where the OSLD has a deeper WED compared to EBT3.

The TPS showed inconsistent measurements largely due to variability in the points selected as skin surface on the TPS, leading to large measurement uncertainty. Other than that, the Eclipse TPS at this centre used PBC algorithm with a dose grid of 2.5 mm x 2.5 mm. The larger voxel size results in volume averaging effect. The inhomogeneity correction used by this algorithm is based on the equivalent tissue air ratio (ETAR) method, which has been found to have a less accurate dose prediction for air cavities and interfaces [33] as well as at the build-up region [34]. The skin sparing effect of 10 MV which lead to the higher dose recorded by 6 MV breast conserving radiotherapy compared to 10 MV for contralateral dose. Radiation dose to the contralateral breast is contributed by scattered radiations from collimator, wedge, and leakage and scatter from the primary radiation [35, 36]. Monitoring of the dose to the contralateral breast is important as these low level radiation doses may induce secondary cancer [37–39].

In patient dose measurement, the results were in good agreement with the phantom study, whereby the OSLD measured higher dose as compared to EBT3 film. Generally, the surface dose in chest wall irradiation was higher compared to breast conserving radiotherapy as the target volume and maximum dose were shifted towards the skin when a 1.0 cm bolus was placed on the skin. The dose differed for every patient due to the difference in breast separations, patient’s set-up and parameter of irradiation. The range of contralateral doses recorded for this study agreed with findings of previous studies by other authors [35, 36, 40]. The total dose to the contralateral breast was found to be below the maximum dose (3.3 Gy) allowed for the contralateral breast, based on the recommendation of the Radiation Therapy Oncology Group (RTOG) breast study protocol [41].

Numerous studies reported that the OSLD has superior physical characteristics for dosimetry such as good reproducibility, linearity [14, 22, 42] and small dependence of energy [43, 44] and dose rate [22]. The OSLD which reaches stability after 16 minutes post-irradiation, has easier and less time consuming readout process compared to films and TLDs [14, 45]. Unlike active dosimeters such as ionization chambers and MOSFET based dosimeter, the OSLDs which are passive dosimeters can be conveniently placed on patients’ body.

## Conclusion

The OSLD was found to be a suitable dosimeter for *in-vivo* dose measurements. However, we have found that the OSLD responds to dose at 0.4 mm depth and thus, consequently overestimates the surface dose. The OSLD can be corrected for surface dose (Hp(0.0)) and skin dose (Hp(0.07)) by multiplying with a factor of 0.42 and 0.54, respectively. In addition, the dose at the depth measured by the dosimeter may be useful in managing late skin toxicity which is due to radiation effects on the skin dermis layer.
